# Effect of probe diffusion on the SOFI imaging accuracy

**DOI:** 10.1038/srep44665

**Published:** 2017-03-23

**Authors:** Wim Vandenberg, Peter Dedecker

**Affiliations:** 1 Department of Chemistry, KU Leuven, Celestijnenlaan 200G, 3001 Heverlee, Belgium

## Abstract

Live-cell super-resolution fluorescence imaging is becoming commonplace for exploring biological systems, though sample dynamics can affect the imaging quality. In this work we evaluate the effect of probe diffusion on super-resolution optical fluctuation imaging (SOFI), using a theoretical model and numerical simulations based on the imaging of live cells labelled with photochromic fluorescent proteins. We find that, over a range of physiological conditions, fluorophore diffusion results in a change in the amplitude of the SOFI signal. The magnitude of this change is approximately proportional to the on-time ratio of the fluorophores. However, for photochromic fluorescent proteins this effect is unlikely to present a significant distortion in practical experiments in biological systems. Due to this lack of distortions, probe diffusion strongly enhances the SOFI imaging by avoiding spatial undersampling caused by the limited labeling density.

Super-resolution or sub-diffraction fluorescence microscopy has opened up a new nanoscale view on life by combining advances in optics, data processing and ‘smart’ fluorophores^1^,^2^. However, the labeled structures are usually assumed to be immobile for the full duration of the imaging, while the fluorophores are considered to be immobile during the period over which they can be observed. A common way to achieve this is to ‘freeze’ the sample dynamics using chemical fixatives. This provides a static picture, if problems such as distortions^3^ and incomplete fixation^4^ can be ruled out. However, this fixation results in the loss of information on dynamics, and as a result sub-diffraction imaging on live cells is becoming increasingly common.

In previous work we and other groups demonstrated that super-resolution optical fluctuation imaging (SOFI) is well suited to live-cell super-resolution imaging^5^,^6^,^7^,^8^, due to its ability to image in challenging conditions such as low signal to noise and high background^9^. The technique operates by analyzing spontaneous fluctuations in fluorophore emission that arise through blinking, made observable by analyzing multiple (100 or more) fluorescence images rapidly acquired from the same sample. Like most sub-diffraction techniques, SOFI provides an improved spatial resolution at the cost of a reduced temporal resolution, though we have recently developed an approach that allows up to a doubling of the acquisition speeds^10^. Importantly, in the original SOFI algorithm as well as later developments, the image formation process can be fully described using an analytical model^11^,^12^,^13^,^14^,^15^. However, this model assumes immobile labels which leads to questions when working in living cells.

Many dynamic processes occur at all times in living systems. For the purpose of this work we consider the cell as an ensemble of supramolecular structures, with fluorophores exhibiting affinity for some of these structures. Motion of the fluorophores can arise in two different ways: the cell structures may move as a whole, taking the labels along with them in a concerted fashion. Examples of this directional motion would be the development of cellular protrusions and organelle motion. On the other hand, the fluorophores can be individually mobile while the structures remain immobile. For example, the plasma membrane can contain microdomains which are immobile on the timescale of the imaging, while the label is free to diffuse and dynamically partition between microdomain and non-microdomain regions, resulting in a heterogeneous steady-state diffusion process. This distinction is shown in Fig. 1.

Directional movement results in a blurring of the sample structure along the path of motion during the image acquisition. How this affects the imaging largely depends on whether the technique uses point-scanning (e.g. STED^16^, RESOLFT^17^, or ISM^18^) or records entire images at once (e.g. PALM^19^, STORM^20^, SIM^21^, or SOFI). The effect of this type of motion has been discussed in detail elsewhere^2^, and in this work we will deal exclusively with the problem of steady-state diffusion. For localization microscopy some initial work has been conducted on this topic^22^. While in the past an approach based on SOFI was used to estimate diffusion rates in real samples^23^, no in-depth analysis of the imaging fidelity has been performed until now. In this contribution we present a detailed investigation of the effect of fluorophore diffusion on the accuracy of SOFI imaging.

## A Theoretical Model for SOFI Imaging in the Presence of Diffusion

We first set out to develop a theoretical description of the SOFI imaging process in the presence of diffusing emitters. We start out by making a number of simplifying assumptions: (i) infinite measurement duration, (ii) no photobleaching of the emitters, (iii) perfect sampling of the emitted fluorescence in space and time, and (iv) all fluorophores have identical spectroscopic properties. These assumptions are in line with the assumptions that have been made in other theoretical models for SOFI^12^,^13^, and serve to make the model analytically tractable.

We assume a conventional imaging model where the fluorescence from a sample is imaged onto a two-dimensional camera detector, such as an EMCCD or sCMOS device. Consider two detector pixels *α* and *β*, located at position *r*_*α*_ and *r*_*β*_. From ref. 13 we know that *XC*_2_(*r*_*α*_, *r*_*β*_), the second order cross-cumulant between these pixels, can be defined as


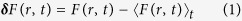



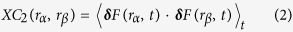


with *F(r, t*) the fluorescence detected at point *r*, and the notation 〈…〉_*t*_ denotes averaging over time. When all the labels are immobile it can be shown that this results in the following signal^13^.


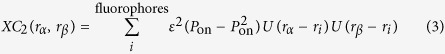


where *U(r*) is the point spread function of the imaging system, *P*_on_ is the on-time-ratio (the fraction of time a fluorophore is in the fluorescent state), and *ε* is the brightness of the fluorophores.

We now limit ourselves to just a single diffusing fluorophore. This implies no loss in generality since the cumulant for multiple fluorophores is just the sum of the cumulants of the individual fluorophores. The fluorescence of a single fluorophore is given by


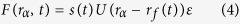


where *s(t*) is a function that is either 1 or 0 depending on whether the molecule is fluorescent at time *t*, and *r*_*f*_ (*t*) is the location of the fluorophore at time *t*. We define the following properties:


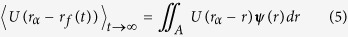



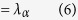


where *ψ(r*) is the probability density of a molecule, meaning that *ψ(r*) provides the probability to observe a fluorophore at a location *r* (which is determined by the sample structuring and the diffusion coefficients in each structure). *A* is the area that is accessible to the fluorophores. We also define


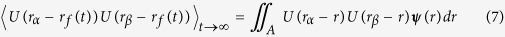



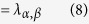



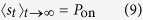


where *P*_on_ is the on-time ratio, that is, the fraction of the time that the fluorophore is in the fluorescent state. Plainly speaking, *λ*_*α*_ is the average fraction of signal from a single fluorophore that can be detected in pixel *α. λ*_*α,β*_ is the average of the product of the fraction that can be detected in pixel *α* and the fraction detected by pixel *β*. Comparing Equations (7) and (3) makes it clear that *λ*_*α,β*_ is proportional to the signal one would expect for a 2nd order SOFI image with perfect sampling. *λ*_*α*_ is merely proportional to a perfectly sampled average image, and for that reason *λ*_*α*_ · *λ*_*β*_ does not contain any super-resolution information.

Given these relations we can expand Equation 2 as follows:


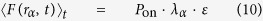



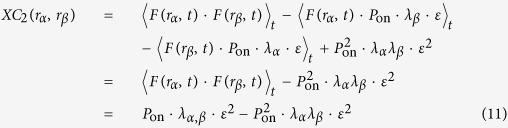


Equation 11 is the main result for the second-order cumulant for a diffusing and blinking molecule. In order to understand this equation we will now consider two extreme situations. We first examine the situation in which there is no diffusion and there are an infinite number of fluorophores, providing perfect sampling. In this case the average contribution of a fluorophore is


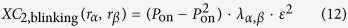


For the second extreme situation we will consider a single molecule that shows diffusion but does not blink (*P*_on_ = 1), with a brightness *ε*′ that is adjusted to the average brightness of the blinking molecule, *ε*′ = *P*_on_*ε*. From equation 11 it can be shown that in this case


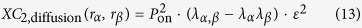


which means that, in general, we can write the expression for a molecule that is blinking and diffusing as


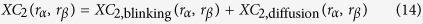


From Equation (13) it is clear that the signal which arises from diffusion is distorted, since it contains a component *λ*_*α*_*λ*_*β*_, while an undistorted and perfectly sampled SOFI image should be should be of the form *c* · *λ*_*α,β*_, with *c* a constant. The fraction of signal coming from diffusion is given by


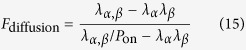


when *P*_on_ is small, which is often the case in actual experiments, this equation reduces to


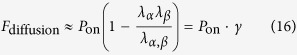


If there is a small separation between pixels *α* and *β* compared to the width of the point spread function, *λ*_*α*_*λ*_*β*_/*λ*_*α,β*_ < 1. This is typically the case for SOFI measurements since the use of cross-cumulants requires a small optical pixel size^13^. In these systems, the contribution of diffusion to the final signal will be limited by this expression.

## Interpreting the Results of the Theoretical Model

Our model shows that the measured SOFI signal *S* is the additive combination of a SOFI signal caused by fluorophore blinking *S*_*B*_ and a signal *S*_*D*_ that arises entirely due to diffusion.


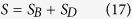


Equation (15) allows us to draw several additional conclusions regarding the magnitude of *S*_*D*_:

1. When *P*_on_ → 1 all signal originates from diffusion, whereas when *P*_on_ → 0 all signal originates from blinking.
2. The relative contribution of diffusion does not depend on the fluorophore brightness.
3. The relative contribution of diffusion does not depend on the kinetics of the fluorescence dynamics.
4. The relative contribution of diffusion does not depend on the rate of diffusion.

In practice, the third and fourth conclusion may not hold when the assumptions of this model are violated. In particular, additional distortions may arise if diffusion is fast enough to cause appreciable displacement during the exposure time of the camera. This point is assessed further along using computer simulations. Similarly, the sample structure may be distorted if the measurement duration is too short for the fluorophores to adequately sample the full sample structure (note that in the worst case this corresponds to a ‘classical’ measurement using immobilized fluorophores). In terms of fluorescence dynamics, the contribution of the blinking to the SOFI signal may also be reduced if these dynamics are very fast or very slow. Blinking that is very fast compared to the camera exposure time will be unobservable, effectively leading to the *P*_on_ → 1 scenario. On the other hand, blinking that is very slow will not be fully captured during the necessarily finite measurement duration, likewise leading to the *P*_on_ → 1 scenario.

The model also predicts that only the component caused by blinking is free of distortions whereas the diffusion component is distorted. Equation (16) shows that the fraction of the signal coming from diffusion is given by


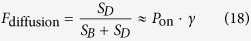


where *P*_on_ is the fraction of the time the fluorophore is in the fluorescent state. *γ* is only dependent on the sample structure and PSF. In realistic situations (sampling of the PSF at the Nyquist limit + SOFI analysis as described in ref. 13) we find that the value of *γ* is approximately 1. As a result, the on-time ratio of the fluorophore places limitation on how strongly the imaging can be distorted by diffusion, with fluorophores with lower on-time ratios (more time spent in the non-fluorescent state) being less susceptible.

In practice, the signal arising through diffusion *S*_*D*_ does not necessarily contain a distorted version of the sample. The degree to which the signal is distorted depends on the specifics of the sample structure. For example, if the combination of sample structure and the pair of pixels being correlated is such that the emission from any single molecule does not give a significant signal in both pixels, while both pixels individually are detecting a significant amount of signal from many molecules, the distortions become more pronounced. However, at the other extreme, if the sample consists of a uniform distribution of fluorophores then the diffusion signal is not distorted. As a result, Equation (18) should be interpreted as an upper bound on the extent to which the SOFI signal is distorted in realistic situations.

While this model clearly illustrates the importance of the on-time ratio in evaluating the effect of diffusion, it does not include several important parameters such as the finite duration of the measurement and the finite exposure times of real camera systems. These effects are hard to include analytically, and in addition it is difficult to evaluate imaging fidelity quantitatively without knowledge of the ground truth sample structure. For these reasons we turned computer simulations based on biological conditions.

## Simulations at Biologically Relevant Conditions

We assumed that the sample consists of a system analogous to membrane microdomains, loosely based on the cholesterol-enriched rafts observed by Mizuno *et al*.^24^, consisting of both rod-like and circular microdomains. The fluorophores undergo free two-dimensional diffusion (reflecting membrane tethering), but with different diffusion coefficient inside and outside the domains. Fluorophores will spontaneously accumulate inside the region with the lowest diffusion coefficient.

While there are many different competing models for membrane microdomain behavior, this simple model reflects a situation where the position of membrane domains is determined by long-lived structuring such as scaffolding by the cytoskeleton^25^. We selected diffusion coefficients inside and outside the microdomains that bracketed values reported in previous studies^26^,^27^. In particular, we performed our simulations using three sets of diffusion coefficients: 0.1 and 1 μm^2^s^−1^, 0.01 and 0.1 μm^2^s^−1^, and 0.1 and 0.2 μm^2^s^−1^. In each case probe diffusion was slower inside the microdomains. (Results for 0.01 and 0.1 μm^2^s^−1^, and 0.1 and 0.2 μm^2^s^−1^ are given in the section ‘Simulations with different diffusion behavior’ in Supplemental Information).

**Figure 2:**
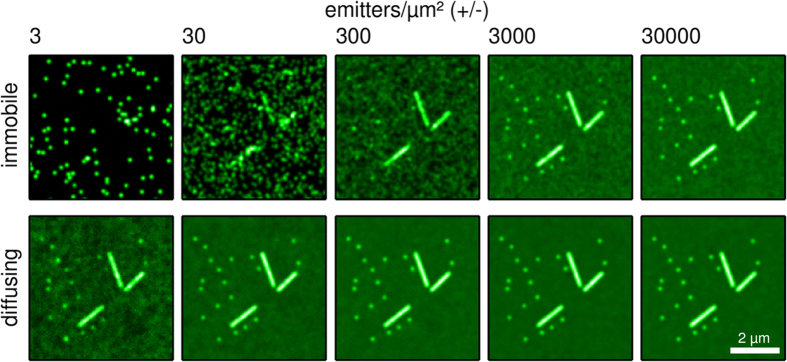
A set of second-order SOFI images where the amount of emitter simulated is varied through 5 orders of magnitude. The emitters are either immobile or diffusing during the experiment. Each image was calculated from 2000 simulated fluorescence images. The ‘diffusing’ case is uniformly rescaled to the same mean intensity as ‘immobile’ case for easy comparison.

Full details on the calculations are provided in the methods section. Briefly, each simulation started with a burn-in period during which the fluorophores were allowed to diffuse, so that a steady-state distribution was established. After this period we generated 2000 simulated fluorescence images, each 30 ms in duration, from which second-order SOFI images were calculated using the Localizer software^28^. The simulations took into account camera noise (including electron multiplication noise), background signal, and photon shot noise. The photochemical behavior was chosen to match typical data recorded using Dronpa as the fluorophore^5^ with an on-time-ratio of 9%, though different conditions such as faster blinking or lower probe brightness where also explored (‘Simulations with different photochemical properties’ in Supplemental Information). For each set of conditions we created two sets of simulated fluorescence images, one in which the positions of the emitters was fixed after the burn in period, and another in which the fluorophores remained mobile. Each simulation was also repeated using different labeling densities, from 3 to 30,000 labels per μm^2^. Figure 2 shows some example images obtained in this way.

## Diffusion Enhances the Spatial Sampling of the Sample Structure

As Fig. 2 shows, spurious sample structuring arises when the emitters are immobile. This spurious structuring is well-known in the imaging community, and arises due to insufficient spatial sampling of the sample structure. Diffusion attenuates this effect because the collective motions of the labels map out the underlying sample structure during the experiment (Supplemental video 1). This finding is not at all unexpected since a similar effect has already been used in PAINT microscopy^29^ and related techniques, and our theoretical model directly predicts improved sampling in the presence of diffusion (Equation 12).

**Figure 3:**
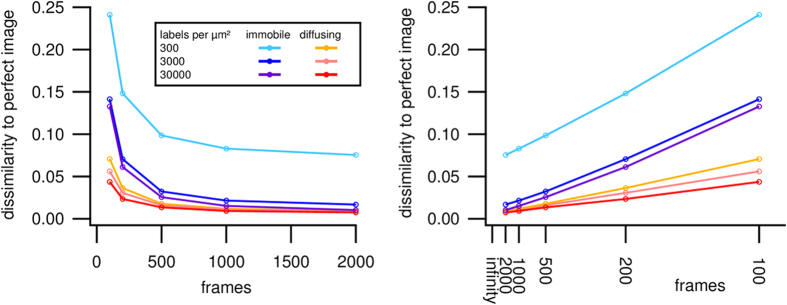
Relative dissimilarity between the SOFI images calculated for the indicated conditions as a function of the number of simulated fluorescence images included in the calculation. On the right side the same data is plotted with the horizontal axis on a reciprocal scale.

Of course, this increased sampling is only beneficial if the diffusion does not introduce significant artifacts. Figure 3 examines this in more detail. This figure shows the dissimilarity of the simulated SOFI image compared to the expected ‘perfect’ SOFI image, for different numbers of emitters and for different numbers of simulated fluorescence images. As a metric for the dissimilarity we used the mean squared error (MSE) over the entire image


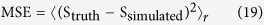


where S is the SOFI signal in each pixel and 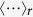 denotes averaging over all pixels in the images. The MSE was in turn averaged over several simulated images (100 images at 300 labels per μm^2^, 10 at 3000 and 1 image at 30000). Because of the computational load, the ground truth was generated using a second simulation of 30000 independently generated immobile emitters per μm^2^.

As is clear from Fig. 3, including more fluorescence images results in a lower dissimilarity, which is simply due to the decreasing noise in the SOFI image. More importantly, this figure shows that the dissimilarity does not trend to zero (perfect match) for immobile emitters, showing that the observed structure is distorted compared to the actual sample structure. The overall dissimilarity is much lower when the emitters are diffusing, even for much lower fluorophore densities.

It is worthwhile to note that previous studies have found physiological concentrations of many membrane-associated molecules in the range of the lower concentrations shown in this figure (receptors densities between 1 and 1000 per μm^2^ at physiological conditions are typical^30^,^31^,^32^).

## Diffusion Causes Negligible Distortion of the SOFI Images

**Figure 4:**
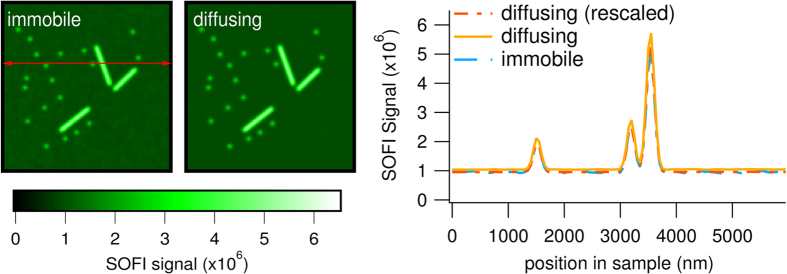
Averages of 100 simulated SOFI images in which the labels are either immobile or diffusing during the experiment. Representative traces are shown, measured at the position of the red arrow in the leftmost panel.

We next sought to quantify the accuracy of the simulated SOFI images with respect to the ground truth sample structure, which requires images that are essentially free of noise. We obtained these by repeating each simulation one hundred times using independent sets of emitters sampled from different burn-in periods. We then calculated an average image of the resulting 100 SOFI images, which results in an image where the effect of the limited spatial sampling and the measurement noise are largely canceled out, and only the systematic bias remains. The images obtained using mobile emitters were then compared to these ground-truth images. An example is shown in Fig. 4.

We observed that diffusion results in slightly higher (about 10%) amplitudes for the SOFI pixels. This is consistent with the used on-time ratio of 9% and the results from our model (Equation 18), which predicts a diffusion-induced component of about this magnitude. Since we are interested in the relative distortions in the image, and not the absolute values, we rescaled the SOFI image with diffusing emitters by a uniform factor to match the reference image as close as possible. After applying this rescaling, there continued to be a relative difference of up to 5% between different regions, which reflects diffusion-induced distortion. Overall this effect is small compared to the expected SNR of a SOFI image^10^ of less than 10, and therefore unlikely to be noticeable in practice. In the section ‘Additive behavior of diffusion and blinking signal’ in Supplementary Information we elaborate on this finding by showing that the total signal can be explained by a mere summation of the signal caused by blinking with the signal caused by diffusion, as our model predicted. We also investigated the effect of the total measurement duration on the deviations (data not shown), and found that the contribution of diffusion to the total signal is slightly larger when less than 50 fluorescence images are included in the calculation. However, this effect is unlikely to be noticeable in practice due to the increased noise in SOFI images calculated from low numbers of fluorescence images.

It is difficult to verify these observations experimentally, as the ground truth sample structure is never known in an actual experiment. Some confirmation can be found in the observations made in ref. 23, where non-blinking beads were used for SOFI imaging. This led to SOFI images in which the observed structure exhibits clear distortions.

## The Camera Exposure Time Determines the Appearance of Distortions

One of the assumptions of the theoretical model is that the there is no movement during the exposure time. As diffusion rates become faster this assumption becomes less and less appropriate, and at some point noticeable artifacts will start to appear. The magnitude of this effect relates to *D* · *t*, where *D* is the diffusion coefficient of the fluorophores and *t* is the exposure time of the camera (the time required to acquire a single fluorescence image). This product determines the distance over which the emitters move in a single fluorescence image. Based on more extensive simulations using different exposure times, we conservatively estimate that distortions due to this mechanism can be neglected as long as *D* · *t* < 0.05 μm^2^ when imaging photochromic fluorescent proteins. For example, with diffusion rates that are an order of magnitude faster than we considered here, such as for cytosolic proteins, no appreciable distortions are to be expected provided that *t* < 5 ms. However, if the probe fluorescence dynamics are such that the blinking-induced signal is much smaller (e.g. almost no emitter blinking), or the fluorophores have higher on-time ratios, then diffusion-induced artifacts are more likely. In that case analogous simulations should be performed to evaluate their impact.

**Figure 5:**
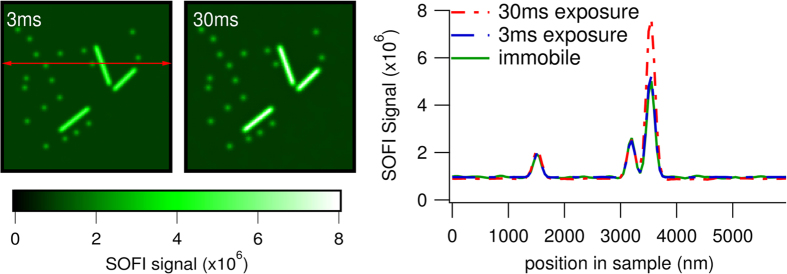
Averages of 100 (3 ms) or 10 (30 ms) simulated SOFI images in which the labels are diffusing during the experiment at diffusion speeds 10-fold higher than in Fig. 4. The exposure time was either 3 or 30 ms as indicated. Both signals were uniformly rescaled to match the ‘immobile’ SOFI image from Fig. 4 which is used as a reference. Representative traces are shown for the 3 and 30 ms case as well as the reference image, measured at the position of the red arrow in the leftmost panel.

To study the effects of much faster diffusion, we repeated the simulation using diffusion coefficients of 1 and 10 μm^2^s^−1^ in the microdomain and non-microdomain regions respectively, in the range of the coefficients observed for cytosolic proteins. When the exposure time is kept at 30 ms noticeable distortions become apparent under these conditions, as is clear when looking at Fig. 5. When the exposure time was reduced in proportion this problem was resolved. This is in line with the explanation given. While 3 ms is a rather short exposure time for most high-performance camera systems, it is a speed which is becoming increasingly more accessible by recent innovations. Alternatively, the light source could be activated in a stroboscopic manner to reduce the effective exposure time while maintaining the same overall acquisition speed.

## Conclusion

In conclusion, our work shows that diffusion of the fluorophores at biologically-relevant diffusion rates and with overall stationary structuring adds a slight bias to the SOFI images. Diffusing emitters also remove the bias caused by observing only a limited number of fluorophores in the sample. We find that, under the conditions considered here, the introduced distortions are small while the effect of improved sampling is substantial. This implies that diffusion has an overall positive effect on the imaging.

## Methods

All simulations were preformed in Igor Pro, WaveMetrics Inc., Lake Oswego, OR, and in purpose written C++ code implemented as a plugin for Igor Pro. SOFI calculations were performed using the Localizer package for Igor which is freely available at https://bitbucket.org/pdedecker/localizer.

The simulations were preformed as described in ref. 5. Briefly, simulated fluorophores were assumed to exist in either a fluorescent or a non-fluorescent state. The lifetimes of each state were assumed to be exponentially distributed, with expectation value 300 ms for the off-state and 30 ms for the on-state except for the data in Supplementary Figure S4). This leads to an on-time-ratio or duty cycle of approximately 9%. These settings where chosen because they closely mirror the behavior of Dronpa, a reversibly switchable fluorescent protein that is commonly used for SOFI imaging. Accordingly, for each simulated fluorophore the time-to-live before switching to the other state was determined by sampling a random number from an exponential distribution with the corresponding expectation value. The camera-exposure time *t* was held fixed at 30 ms.

To model diffusion, each simulated fluorescence image was subdivided into a number of discrete time steps where the effect of diffusion was assumed to be negligible. The length of these stretches is given by 300 μs divided by the fastest diffusion coefficient used during the simulation (in μm^2^s^−1^). Using this formulation, the simulation time step ranged from 30 μs to 3 ms, corresponding to between 1000 and 10 time steps per simulated fluorescence image.

At each time step, the position of all fluorophores was updated by a distance *D* · *N*(4*δ*), where *D* is the diffusion coefficient, *δ* is the simulation time step, and *N(σ*^2^) represents the sampling of a normal distribution with variance *σ*^2^. The sample was considered to have toroidal geometry (meaning the diffusing molecules ‘wrapped around’ to the other side if they would leave the simulated area). To avoid the introduction of distortions through this wrapping mechanism, molecules were allowed to diffuse over an area of 9 · *A*, where *A* is the area that is visible in the simulated camera, located at the center of this total simulated area.

At each simulation time step, the coordinates of fluorophores in the fluorescent state were stored in a separate dataset. For each simulated fluorescence image, the coordinates of all active fluorophores at every simulation timestep within that exposure time were treated as independent emitters with total number of emitted photons *εt*. We assumed a Gaussian PSF with a standard deviation of 100 nm, identical to the optical pixel size, and the emission of each emitter was integrated over the area of the simulated detector pixel. Shot noise was modeled by replacing the total number of photons in each pixel with a random value drawn from a Poisson distribution with the same expectation value. Finally, noise arising due to the electron multiplication process was included based on the procedure described by Ulbrich and Isacoff^33^.

Before the actual experiment a burn-in period was implemented, in which the molecules could achieve their steady-state distribution. The length of this burn-in period was chosen by a control experiment, in which the evolution of the fluorescence of the image was followed. At the beginning of this experiment there was an exponential rise in the overall fluorescence emitted from the sample. The burn-in period for the actual simulations was chosen to be at least 5 times longer than the half-life of this exponential rise. This resulted in 750 burn-in images when the diffusion coefficients were 0.1/1 μm^2^s^−1^, 6,000 for 0.01/0.1 μm^2^s^−1^ and 2,500 for 0.1/0.2 μm^2^s^−1^.

## Additional Information

**How to cite this article:** Vandenberg, W. and Dedecker, P. Effect of probe diffusion on the SOFI imaging accuracy. *Sci. Rep.* **7**, 44665; doi: 10.1038/srep44665 (2017).

**Publisher's note:** Springer Nature remains neutral with regard to jurisdictional claims in published maps and institutional affiliations.

## Supplementary Material

Supplementary Video 1

Supplementary Information

## Figures and Tables

**Figure 1:**
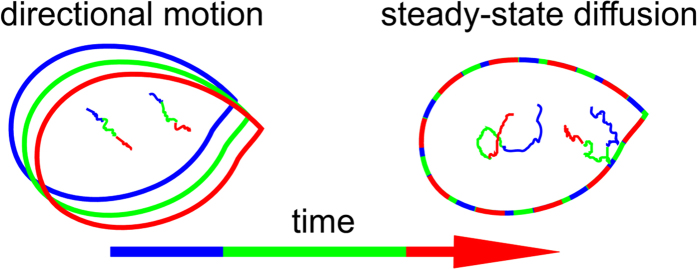
Two different categories of movement. A structure is shown at three time-points during acquisition (blue to green to red), Example trajectories of two fluorophores within this structure are shown in matching colors.

## References

[b1] Dedecker P., De Schryver F. C. & Hofkens J. Fluorescent proteins: shine on, you crazy diamond. J. Am. Chem. Soc. 135, 2387–2402 (2013). 23317378 10.1021/ja309768d

[b2] Vandenberg W., Leutenegger M., Lasser T., Hofkens J. & Dedecker P. Diffraction-unlimited imaging: from pretty pictures to hard numbers. Cell Tissue Res. 360, 151–178 (2015). 25722085 10.1007/s00441-014-2109-0

[b3] Ryter A. Contribution of new cryomethods to a better knowledge of bacterial anatomy. Ann. Inst. Pasteur Microbiol. 139, 33–44 (1988). 10.1016/0769-2609(88)90095-6 3289587

[b4] Tanaka K. A. . Membrane molecules mobile even after chemical fixation. Nat. Methods 7, 865–866 (2010). 20881966 10.1038/nmeth.f.314

[b5] Dedecker P., Mo G. C., Dertinger T. & Zhang J. Widely accessible method for superresolution fluorescence imaging of living systems. Proc. Natl. Acad. Sci. USA 109, 10909–10914 (2012). 22711840 10.1073/pnas.1204917109 PMC3390831

[b6] Geissbuehler S. . Live-cell multiplane three-dimensional super-resolution optical fluctuation imaging. Nat Commun 5, 5830 (2014). 25518894 10.1038/ncomms6830 PMC4284648

[b7] Duwe S. . Expression-Enhanced Fluorescent Proteins Based on Enhanced Green Fluorescent Protein for Super-resolution Microscopy. ACS Nano 9, 9528–9541 (2015). 26308583 10.1021/acsnano.5b04129

[b8] Girsault A. . SOFI Simulation Tool: A Software Package for Simulating and Testing Super-Resolution Optical Fluctuation Imaging. PLoS One 11, e0161602 (2016). 27583365 10.1371/journal.pone.0161602 PMC5008722

[b9] Geissbuehler S. . Comparison between SOFI and STORM. Biomed Opt Express 2, 408-420 (2011). 21412447 10.1364/BOE.2.000408 PMC3047347

[b10] Vandenberg W. . Model-free uncertainty estimation in stochastical optical fluctuation imaging (SOFI) leads to a doubled temporal resolution. Biomed Opt Express 7, 467–480 (2016). 26977356 10.1364/BOE.7.000467 PMC4771465

[b11] Deschout H. . Complementarity of PALM and SOFI for super-resolution live-cell imaging of focal adhesions. Nat commun. 7, 13693 (2016). 27991512 10.1038/ncomms13693 PMC5187410

[b12] Dertinger T., Colyer R., Iyer G., Weiss S. & Enderlein J. Fast, background-free, 3D super-resolution optical fluctuation imaging (SOFI). Proc. Natl. Acad. Sci. USA 106, 22287–22292 (2009). 20018714 10.1073/pnas.0907866106 PMC2799731

[b13] Dertinger T., Colyer R., Vogel R., Enderlein J. & Weiss S. Achieving increased resolution and more pixels with Superresolution Optical Fluctuation Imaging (SOFI). Opt Express 18, 18875–18885 (2010). 20940780 10.1364/OE.18.018875 PMC3072111

[b14] Stein S. C., Huss A., Hahnel D., Gregor I. & Enderlein J. Fourier interpolation stochastic optical fluctuation imaging. Opt Express 23, 16154–16163 (2015). 26193588 10.1364/OE.23.016154

[b15] Geissbuehler S. . Mapping molecular statistics with balanced super-resolution optical fluctuation imaging (bSOFI)). Opt Nanoscopy 1 (2012).

[b16] Klar T. A. & Hell S. W. Subdiffraction resolution in far-field fluorescence microscopy. Opt Lett 24, 954–956 (1999). 18073907 10.1364/ol.24.000954

[b17] Hofmann M., Eggeling C., Jakobs S. & Hell S. W. Breaking the diffraction barrier in fluorescence microscopy at low light intensities by using reversibly photoswitchable proteins. Proc. Natl. Acad. Sci. USA 102, 17565–17569 (2005). 16314572 10.1073/pnas.0506010102 PMC1308899

[b18] Muller C. B. & Enderlein J. Image scanning microscopy. Phys. Rev. Lett. 104, 198101 (2010). 20867000 10.1103/PhysRevLett.104.198101

[b19] Betzig E. . Imaging intracellular fluorescent proteins at nanometer resolution. Science 313, 1642–1645 (2006). 16902090 10.1126/science.1127344

[b20] Rust M. J., Bates M. & Zhuang X. Sub-diffraction-limit imaging by stochastic optical reconstruction microscopy (STORM). Nat. Methods 3, 793–795 (2006). 16896339 10.1038/nmeth929 PMC2700296

[b21] Gustafsson M. G. Surpassing the lateral resolution limit by a factor of two using structured illumination microscopy. J Microsc 198, 82–87 (2000). 10810003 10.1046/j.1365-2818.2000.00710.x

[b22] Deschout H., Neyts K. & Braeckmans K. The influence of movement on the localization precision of sub-resolution particles in fluorescence microscopy. J Biophotonics 5, 97–109 (2012). 22083848 10.1002/jbio.201100078

[b23] Kisley L. . Characterization of Porous Materials by Fluorescence Correlation Spectroscopy Super-resolution Optical Fluctuation Imaging. ACS Nano 9, 9158–9166 (2015). 26235127 10.1021/acsnano.5b03430 PMC10706734

[b24] Mizuno H. . Fluorescent probes for superresolution imaging of lipid domains on the plasma membrane. Chem Sci 2, 1548–1553 (2011).

[b25] Gomez-Llobregat J., Buceta J. & Reigada R. Interplay of cytoskeletal activity and lipid phase stability in dynamic protein recruitment and clustering. Sci Rep 3, 2608 (2013). 24018870 10.1038/srep02608 PMC3767946

[b26] Lommerse P. H., Snaar-Jagalska B. E., Spaink H. P. & Schmidt T. Single-molecule diffusion measurements of H-Ras at the plasma membrane of live cells reveal microdomain localization upon activation. J. Cell. Sci. 118, 1799–1809 (2005). 15860728 10.1242/jcs.02300

[b27] Lu S. . The spatiotemporal pattern of Src activation at lipid rafts revealed by diffusion-corrected FRET imaging. PLoS Comput. Biol. 4, e1000127 (2008). 18711637 10.1371/journal.pcbi.1000127 PMC2517613

[b28] Dedecker P., Duwe S., Neely R. K. & Zhang J. Localizer: fast, accurate, open-source, and modular software package for superresolution microscopy. J Biomed Opt 17, 126008 (2012). 23208219 10.1117/1.JBO.17.12.126008 PMC3512108

[b29] Sharonov A. & Hochstrasser R. M. Wide-field subdiffraction imaging by accumulated binding of diffusing probes. Proc. Natl. Acad. Sci. USA 103, 18911–18916 (2006). 17142314 10.1073/pnas.0609643104 PMC1748151

[b30] Nieuwenhuizen R. P. . Quantitative localization microscopy: effects of photophysics and labeling stoichiometry. PLoS One 10, e0127989 (2015). 25992915 10.1371/journal.pone.0127989 PMC4439177

[b31] Wilma van Esse G. . Quantification of the brassinosteroid insensitive1 receptor in planta. Plant Physiol. 156, 1691–1700 (2011). 21617031 10.1104/pp.111.179309 PMC3149942

[b32] Chen Y. . Mapping receptor density on live cells by using fluorescence correlation spectroscopy. Chemistry 15, 5327–5336 (2009). 19360825 10.1002/chem.200802305 PMC5525150

[b33] Ulbrich M. H. & Isacoff E. Y. Subunit counting in membrane-bound proteins. Nat. Methods 4, 319–321 (2007). 17369835 10.1038/NMETH1024 PMC2744285

