# Supplementary material for: Effect of probe diffusion on the SOFI imaging accuracy
Source: Sci Rep. 2017 Mar 23;7:44665. doi: 10.1038/srep44665 (PMC5363082; doi:10.1038/srep44665)
Supplement: Supplementary Information [file srep44665-s2.pdf]

Effect of probe diffusion on the SOFI imaging accuracy
Supporting information

Wim Vandenberg and Peter Dedecker

Specifics of the simulated structure

The structure that was simulated corresponds to the map of local diffusion coefficients shown on the left of figure S1, in the middle of this figure the corresponding average wide-field image is shown (calculated using a simulation of 2000 frames using 45000 emitters). A trace at the location of the red arrow is shown on the right. The circular rafts that are simulated have a diameter of 100 nm. The elongated rafts are defined as a certain distance (50 nm) to a line segment, leading to a short axis of 100 nm.

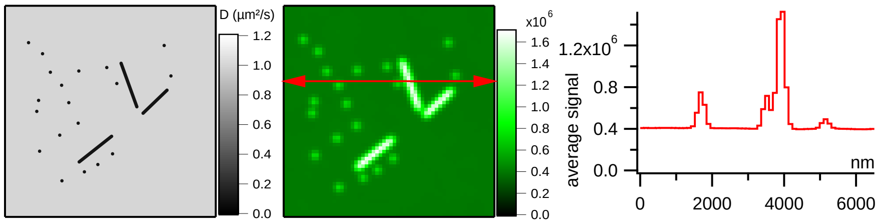

Figure S1: The structure of the rafts used in this manuscript. On the leftmost side an image of the local diffusion coefficient is shown. In the middle a 'diffraction limited´ average image, and on the right a trace through this average image are shown.

Simulations with different diffusion behavior

In this section results are shown for combinations of diffusion coefficients of 0.01 and 0.1 $\mu m^2\,s^{-1}$ as well as 0.1 and 0.2 $\mu m^2\,s^{-1}$ for microdomain and non-microdomain regions respectively, representing a slower diffusion and a smaller ratio of diffusion coefficients compared to the main text. When we evaluate the effect of probe concentrations at these diffusion speeds we see essentially the same behavior as shown in figure 2 in main text. However, the additional sampling in the case of slower diffusion is somewhat reduced as can be seen in Supplementary Figure S2. This is expected since the at these reduced speeds the molecules can no longer sample the entire image during the recorded 2000

frames, clearly breaking the assumption of an infinite measurement present in the theoretical model.

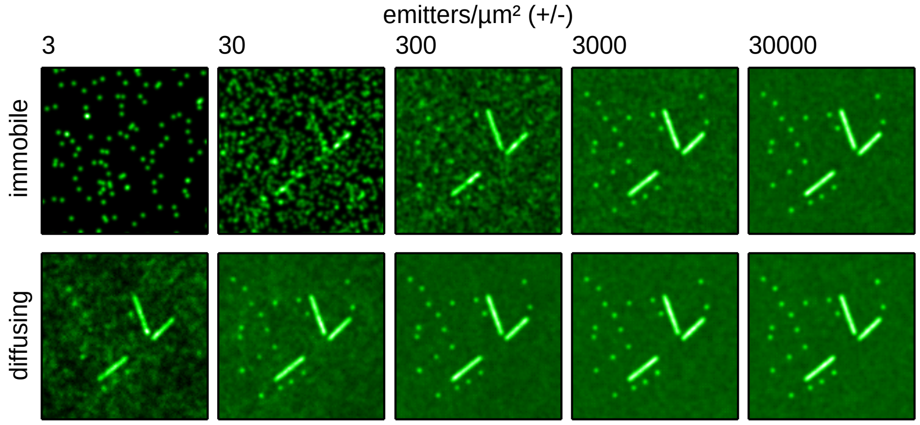

Figure S2: A set of second-order SOFI images where the amount of emitter simulated is varied through 5 orders of magnitude. In this figure diffusion is 10-fold slower compared to figure 2 in main text. Each image was calculated from 2000 simulated fluorescence images. The 'diffusing' case is uniformly rescaled to the same mean intensity as 'immobile' case for easy comparison.

To check for systematic bias we averaged 100 SOFI images created from independent simulations as in main text. Despite the fact that the image made with a smaller ratio of diffusion speeds is somewhat more noisy, no noticeable differences between diffusing and immobile scenarios could be found, as can be seen in Supplementary Figure S3.

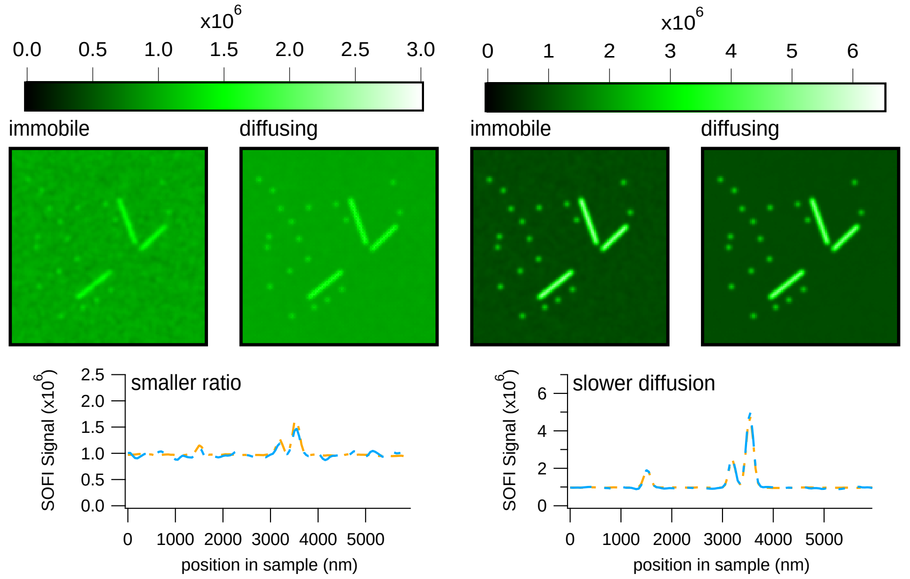

Figure S3: Averages of 100 simulated SOFI images in which the labels are either immobile or diffusing during the experiment. 'smaller ratio' indicates that diffusion in the non-raft regions was 5-fold slower compared to figure 4 in main text, 'slower diffusion' indicates that all diffusion coefficients where decreased by a factor of 10 compared to figure 4 in main text. Traces trough the images are shown with immobile emitters in orange, and diffusing emitters in blue.

Simulations with different photochemical properties

In this section we evaluated the influence of the switching speed and the intrinsic brightness of the fluorophore on the fidelity of the imaging. For the 'reduced brightness' case we reduced the brightness from 10,000 photons s^{-1} in previous simulations to 1,000 photons s^{-1}, for the 'faster switching' case the mean time a molecule spends in the on and off state was reduced by a factor of 5, making it 6 and 60 ms respectively. The diffusion coefficients were 0.1 and 1 $\mu m^2\,s^{-1}$ for microdomain and non-microdomain regions respectively as in main text. No distortions where found in either case, as is visualized in Supplementary Figure S4.

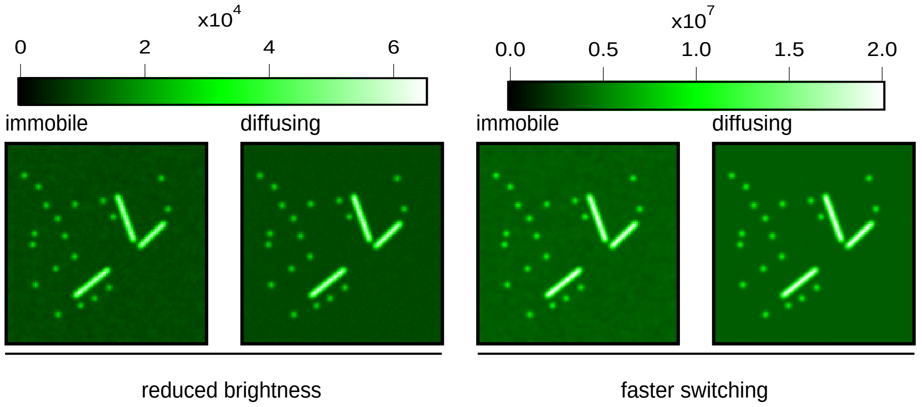

Figure S4: Averages of 100 simulated SOFI images in which the labels are either immobile or diffusing during the experiment. 'Reduced brightness' implies a 10-fold reduction in brightness compared to figure 4 in main text, Faster switching implies reduction of the residence time of both on and off state by a factor of 5 . The 'diffusing' case is uniformly rescaled to the same mean intensity as 'immobile' case for easy comparison.

Additive behavior of diffusion and blinking signal

To prove that the signal can be decomposed in a diffusion and a blinking component, as the model predicted, we repeated these experiments with diffusing but non-blinking (on-time ratio 100%) emitters. The brightness of these emitters was reduced to match the same average brightness per molecule as in the previous calculations. We observed that the signal obtained in this way matched the difference between experiments performed with diffusing and immobile labels as predicted. This is shown in Supplementary Figure S5.

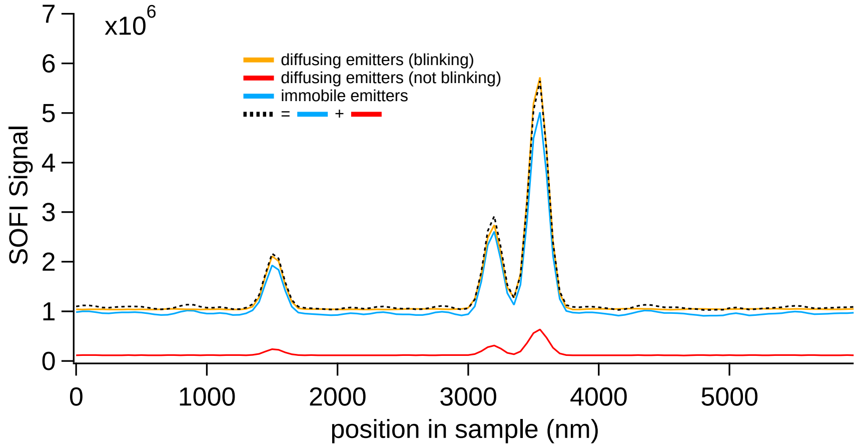

Figure S5: Traces through average simulated SOFI images (50 images averaged in case of 'not blinking', 100 for all other cases). In orange the emitters were blinking and diffusing during the experiment, in blue they were only blinking but not diffusing and in red they were diffusing but not blinking. The black dotted line shows the sum of the red and the blue line.

Movie legend

Supplemental Video S1: Diffusion of molecules during acquisition
In this movie on the left diffusing emitters are shown for reference, while a 'cumulative signal' is shown on the right. This movie corresponds to 2000 frames of recorded data. The simulated emitters follow the same diffusion behavior as in figure 2 in main text (0.1 and 1 $\mu m^2\,s^{-1}$), but do not blink, emit 10 times more photons and are present at a concentration 10 times lower than the lowest concentration shown in this figure.
